# Supplementary material for: Engineering mouse cell fate controller by rational design
Source: Nat Commun. 2024 Jul 23;15:6200. doi: 10.1038/s41467-024-50551-2 (PMC11266670; doi:10.1038/s41467-024-50551-2)
Supplement: Supplementary file 1 — Supplementary Information [file 41467_2024_50551_MOESM1_ESM.pdf]

Figure S1

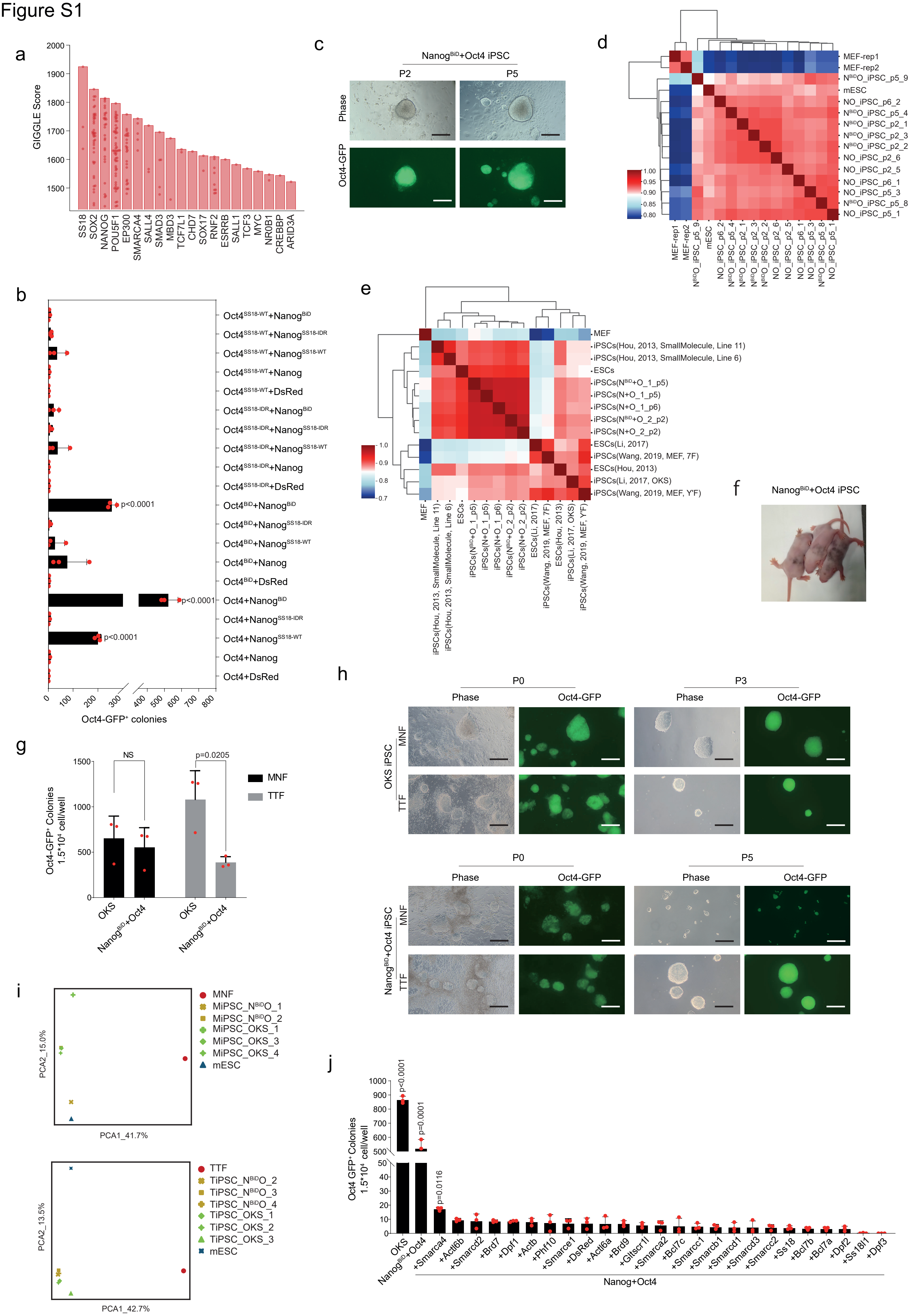

a
GIGGLE Score
SS18 SOX2 NANOG POU5F1 EP300 SMARCA4 SALL4 SMAD3 MBD3 TCF7L1 CHD7 SOX17 RNF2 ESRRB SALL1 TCF3 MYC NR0B1 CREBBP ARID3A
b
Oct4-GFP+ colonies
p<0.0001
c
NanogBiD+Oct4 iPSC
P2
P5
Phase
Oct4-GFP
d
e
f
NanogBiD+Oct4 iPSC
g
Oct4-GFP+ Colonies 1.5*10^4 cell/well
NS
p=0.0205
MNF
TTF
OKS
NanogBiD+Oct4
h
P0
P3
P5
Phase
Oct4-GFP
OKS iPSC
NanogBiD+Oct4 iPSC
MNF
TTF
i
PCA1_41.7%
PCA2_15.0%
PCA1_42.7%
PCA2_13.5%
mESC
j
Nanog+Oct4

Supplementary figure 1. Nanog^{BiD} facilitates somatic cell reprogramming.

a. Bar plots show the top 20 correlated factors with GIGGLE scores measuring the correlation of each factor's binding site with the ESC mESC-specific open chromatin loci.

b. Numbers of Oct4-GFP positive colonies induced after SS18 fusion protein infection on day 12. Bars are mean with SD, and red plots are individual data points for n=3 biological replicates. Statistical significance relevant to number of Oct4-GFP$^+$ colonies of *Nanog+Oct4* was measured with two-tailed unpaired *t*-test. P values were performed on each graph.

c. Morphology representative of an iPSC line induced with Nanog^{BiD}+*Oct4* at passage 2 and passage 5. Scale bar, 250 µm.

d and **e.** The R2 correlation coefficient matrix of all versus all samples and all RNA-seq datasets as indicated. NO indicates *Nanog+Oct4*. N^{BiD}O indicates Nanog^{BiD}+*Oct4*.

f. Chimeric mice of Nanog^{BiD}+*Oct4* iPSCs.

g. Number of iPSC colonies induced from MNFs and TTFs infected by Nanog^{BiD}+*Oct4* or OKS on day 12. Data are presented as mean ± SD (n=3 biological replicates), *p* values are determined by two-tailed unpaired *t*-test and performed on each graph. NS, nonsignificant.

h. Represented images of iPSCs from MNFs and TTFs. Scale bar, 250 µm.

i. PCA analysis of RNA-seq data of mESC, and iPSCs induced from MNFs and TTFs infected by Nanog^{BiD}+*Oct4* or OKS. NO indicates *Nanog+Oct4*. N^{BiD}O indicates Nanog^{BiD}+*Oct4*. MiPSC indicates iPSC derived from MNFs. TiPSC indicates iPSC derived from TTFs.

j. The effects of BAF subunits in *Nanog+Oct4* mediated reprogramming system. Data are presented as mean ± SD (n=3 biological replicates), *p* values are determined by two-tailed unpaired *t*-test and performed on each graph.

Figure S1 related to Figure 1. Source data are provided as a Source Data file.

Figure S2

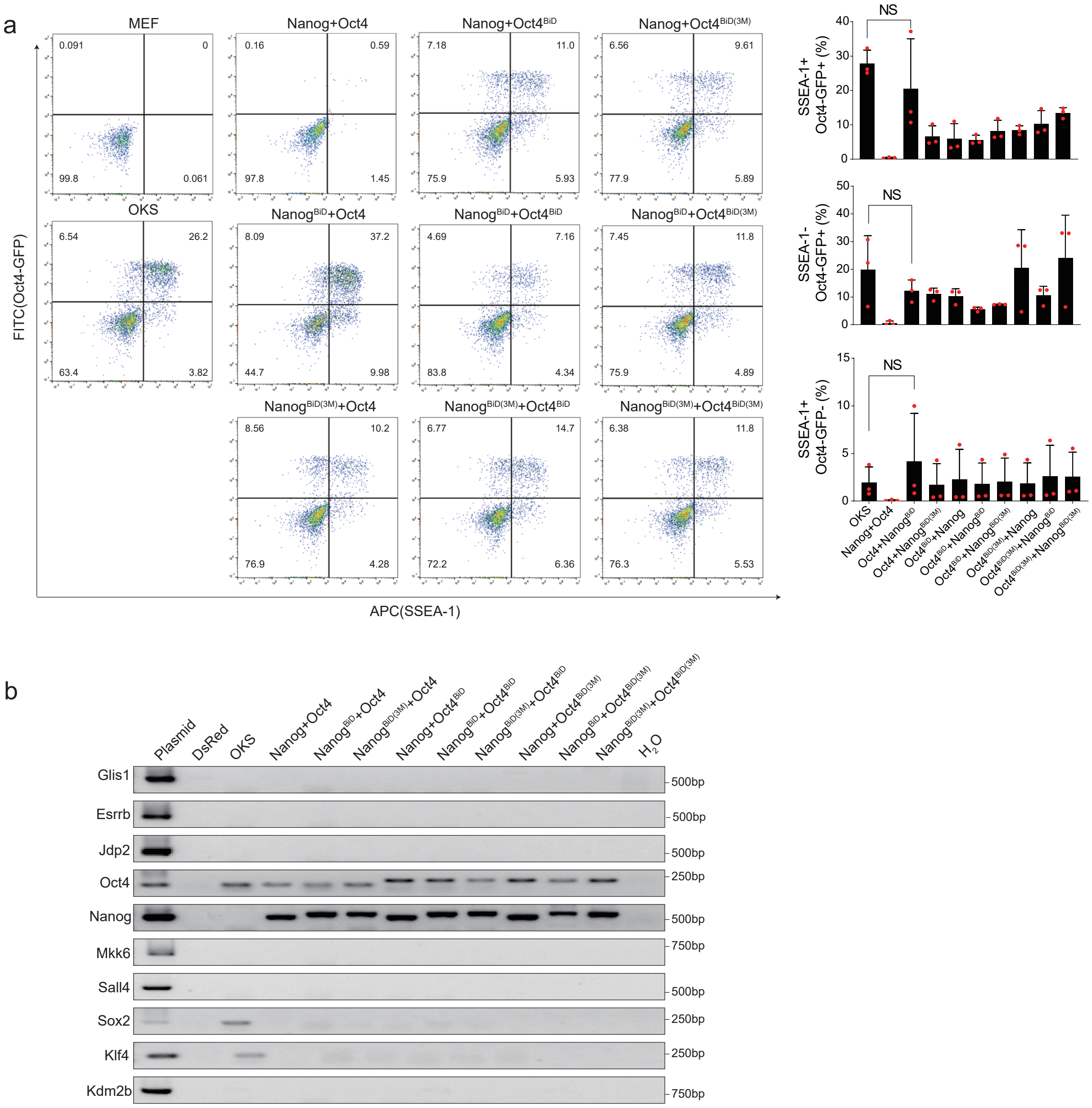

Supplementary figure 2. Nanog^{BiD} enhance reprogramming efficiency.

a. Flow cytometric analysis for the expression of SSEA1 and Oct4-GFP in colonies on day 12 after reprogramming. Data are presented as mean ± SD (n=3 biological replicates) and *p* values are determined by two-tailed unpaired *t*-test. NS, nonsignificant.

b. Detection of plasmid integration by PCR. Gels shown are representative of n=2 independent experiments.

Figure S2 related to Figure 2. Source data are provided as a Source Data file.

Figure S3

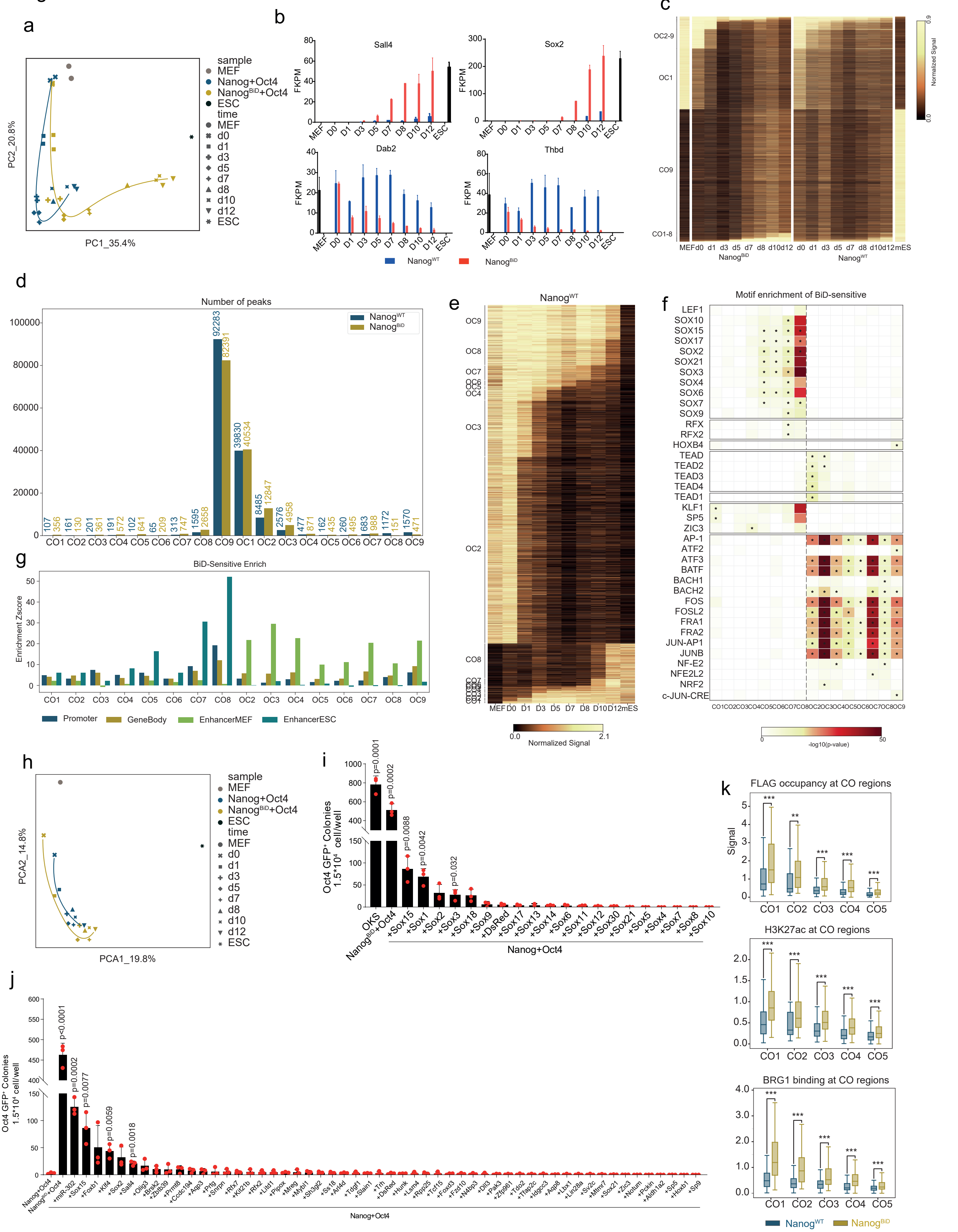
a
b
c
d
e
f
g
h
i
j
k
PC1_35.4%
PC2_20.8%
sample
MEF
Nanog+Oct4
Nanog^{BiD}+Oct4
ESC
time
d0
d1
d3
d5
d7
d8
d10
d12
Sall4
Sox2
Dab2
Thbd
FKPM
Nanog^{WT}
Nanog^{BiD}
Normalized Signal
Number of peaks
BiD-Sensitive Enrich
Enrichment Zscore
Promoter
GeneBody
EnhancerMEF
EnhancerESC
Motif enrichment of BiD-sensitive
-log10(p-value)
PCA1_19.8%
PCA2_14.8%
Oct4 GFP^{+} Colonies 1.5*10^{4} cell/well
Nanog+Oct4
FLAG occupancy at CO regions
H3K27ac at CO regions
BRG1 binding at CO regions
Signal
CO1
CO2
CO3
CO4
CO5

Supplementary figure 3. Nanog^{BiD} plays a major role in opening chromatin.

a. Principal Component Analysis of all genes' expression. Samples are colored by different cell types and reprogramming paths. Different shapes indicate different reprogramming stages.

b. Bar plots show the expression dynamics of selected genes during the Nanog^{WT} and Nanog^{BiD} reprogramming path as well as MEF and ESC.

c. Heatmap of the chromatin accessibility dynamics during Nanog^{WT} and Nanog^{BID} reprogramming path as well as MEF and mES. Loci of open chromatin changed from closed to open (CO) or open to closed (OC).

d. Bar plots of the peak number of each CO and OC loci as defined in figure 3e.

e. Heatmap of the chromatin accessibility dynamics during Nanog^{WT} reprogramming path as well as MEF and ESC. Loci of open chromatin changed from closed to open (CO) or open to closed (OC).

f. Motif of transcription factors significantly enriched in each group of the Nanog^{BiD} -Sensitive regions as defined in Figure 3g. Motifs with at least 3-fold enrichment and less than $1\times10^{\wedge-5}$ p-value are marked with asterisk.

g. Bar plots of the Nanog^{BiD}-Sensitive chromatin accessibility dynamics enrichments in promoter, genebody, enhancerMEF, and enhancerESC. The enhancer regions were downloaded from EnhancerAtlas (MEF_E13.5, ESC_D0).

h. PCA of all ATAC-seq peaks. Samples are colored by different cell types and reprogramming paths. Different shapes indicate different reprogramming stages.

i and **j.** The effects of Sox family transcription factors or selected genes in *Nanog+Oct4* mediated reprogramming system. Data are presented as mean ± SD (n=3 biological replicates), *p* values are determined by two-tailed unpaired *t*-test and performed on each graph.

k. NANOG/H3K27ac/BRG1 CUT&Tag signal in each group of the Nanog^{BiD}-Sensitive regions (defined in Fig 3g). The box plots indicate the medians (centerlines), first and third quartiles (bounds of boxes) and 1.5 multiply by interquartile range (whiskers). Statistical analysis was performed using student's two-sided *t*-test. ***p<0.0001, **p<0.01, Precise *p* values were provided in the Source Data file.

Figure S3 related to Figure 3. Source data are provided as a Source Data file.

Figure S4

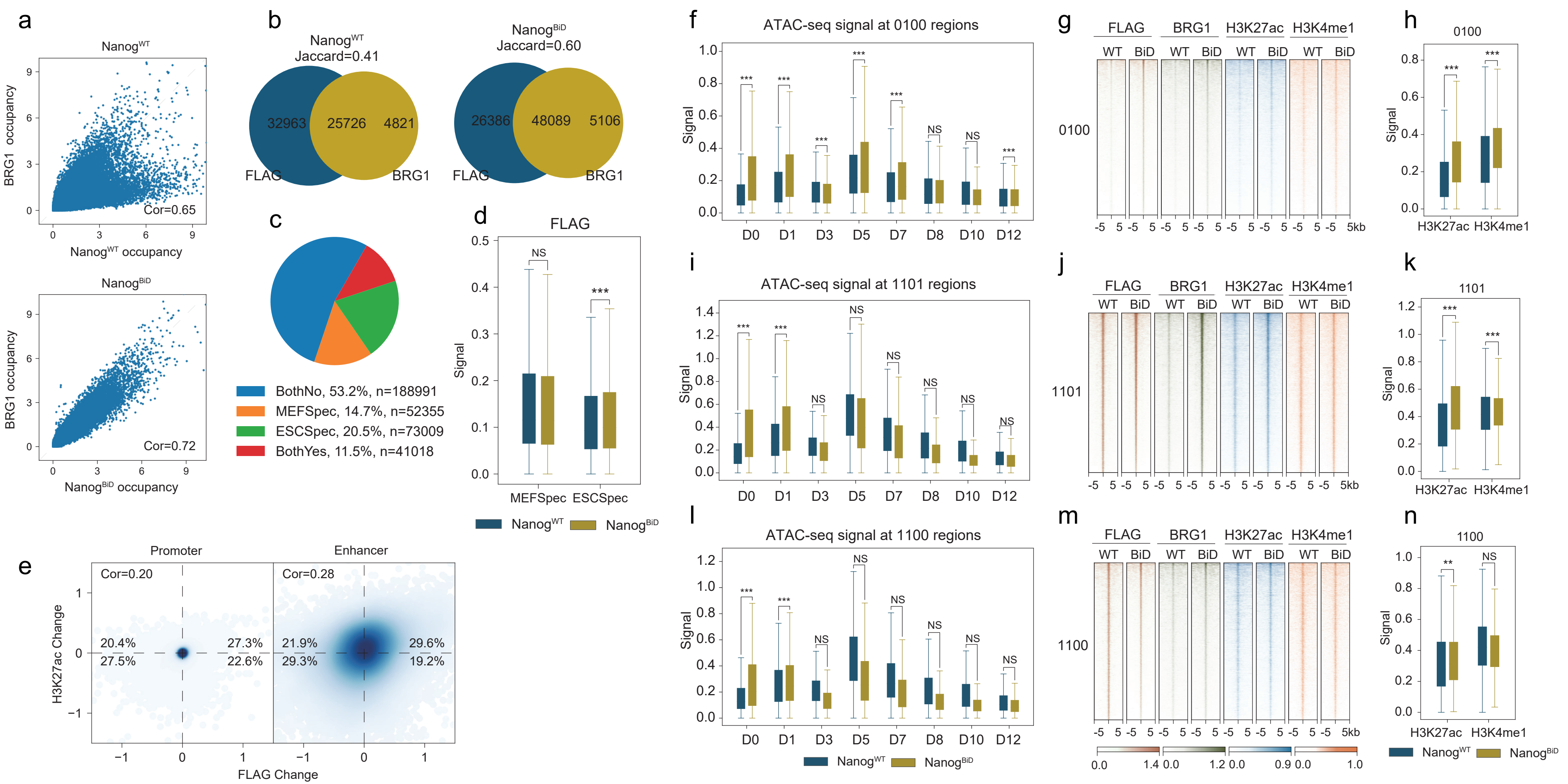

a
NanogWT
BRG1 occupancy
NanogWT occupancy
Cor=0.65
NanogBiD
NanogBiD occupancy
Cor=0.72
b
NanogWT
Jaccard=0.41
32963
25726
4821
FLAG
BRG1
NanogBiD
Jaccard=0.60
26386
48089
5106
c
BothNo, 53.2%, n=188991
MEFSpec, 14.7%, n=52355
ESCSpec, 20.5%, n=73009
BothYes, 11.5%, n=41018
d
FLAG
Signal
MEFSpec
ESCSpec
NanogWT
NanogBiD
e
Promoter
Enhancer
Cor=0.20
Cor=0.28
20.4%
27.5%
27.3%
22.6%
21.9%
29.3%
29.6%
19.2%
H3K27ac Change
FLAG Change
f
ATAC-seq signal at 0100 regions
i
ATAC-seq signal at 1101 regions
l
ATAC-seq signal at 1100 regions
D0
D1
D3
D5
D7
D8
D10
D12
g
j
m
FLAG
BRG1
H3K27ac
H3K4me1
WT
BiD
0100
1101
1100
5kb
h
k
n
H3K27ac
H3K4me1

Supplementary Figure 4. Nanog^{BiD} and BRG1 co-occupancy at Nanog^{BiD}-specific regions.

a. The consistency of NANOG and BRG1 binding at D5 in Nanog^{WT} and Nanog^{BiD}. Each point represents the average signal across the 1000bp genome region of NANOG and BRG1 CUT&Tag signal. The spearman correlation coefficients were calculated.

b. The overlap peaks of NANOG and BRG1 binding at D5 in two reprogramming paths.

c. The numbers of four different types of NANOG motif. MEF Spec indicated the motif are specifically opened at the MEF stage. ESC Spec indicated the motif are is specifically opened at ESC stage. Both Yes indicated the motif are opened at both MEF and ESC stage. Both No indicated the motif are is closed at both MEF and ESC stage.

d. Boxplot show the NANOG CUT&Tag signal of MEF Spec and ESC Spec motifs.

e. The relationship of NANOG binding change and H3K27ac binding change in the promoter regions and enhancer regions. The spearman correlation coefficients were calculated for each type of region.

f, i, and l. Boxplot of the ATAC-seq signal at each stage of Nanog^{WT} and Nanog^{BiD} reprogramming path in 0100, 1101 and 1100 regions.

g, j, and m. Heatmap of the NANOG binding signal, BRG1 binding signal, H3K27ac signal, and H3K4me1 signal at D5 of Nanog^{WT} and Nanog^{BiD} reprogramming path in 0100, 1101 and 1100 regions.

h, k, and n. Boxplot of the H3K27ac signal, and H3K4me1 signal at D5 of Nanog^{WT} and Nanog^{BiD} reprogramming path in 0100, 1101 and 1100 regions.

The box plots (**f**,**h**,**i**,**k**,**l**,**n**) indicate the medians (centerlines), first and third quartiles (bounds of boxes) and 1.5 multiply by interquartile range (whiskers). Statistical analysis was performed using student's two-sided *t*-test. ***p<0.0001, **p<0.01, Precise *p* values were provided in the Source Data file. NS, nonsignificant. WT indicates Nanog^{WT}. BiD indicates Nanog^{BiD}. Figure S4 related to Figure 4. Source data are provided as a Source Data file.
