# Supplementary material for: Engineering mouse cell fate controller by rational design
Source: Nat Commun. 2024 Jul 23;15:6200. doi: 10.1038/s41467-024-50551-2 (PMC11266670; doi:10.1038/s41467-024-50551-2)
Supplement: Supplementary file 3 — Reporting summary [file 41467_2024_50551_MOESM3_ESM.pdf]

nature portfolio

Corresponding author(s): Duanqing Pei, Bo Wang, Chengchen Zhao

Last updated by author(s): 2024-6-24

Reporting Summary

Nature Portfolio wishes to improve the reproducibility of the work that we publish. This form provides structure for consistency and transparency in reporting. For further information on Nature Portfolio policies, see our Editorial Policies and the Editorial Policy Checklist.

Statistics

For all statistical analyses, confirm that the following items are present in the figure legend, table legend, main text, or Methods section.

n/a	Confirmed	
☐	☒	The exact sample size (*n*) for each experimental group/condition, given as a discrete number and unit of measurement
☐	☒	A statement on whether measurements were taken from distinct samples or whether the same sample was measured repeatedly
☐	☒	The statistical test(s) used AND whether they are one- or two-sided<br>*Only common tests should be described solely by name; describe more complex techniques in the Methods section.*
☒	☐	A description of all covariates tested
☒	☐	A description of any assumptions or corrections, such as tests of normality and adjustment for multiple comparisons
☐	☒	A full description of the statistical parameters including central tendency (e.g. means) or other basic estimates (e.g. regression coefficient) AND variation (e.g. standard deviation) or associated estimates of uncertainty (e.g. confidence intervals)
☒	☐	For null hypothesis testing, the test statistic (e.g. *F*, *t*, *r*) with confidence intervals, effect sizes, degrees of freedom and *P* value noted<br>*Give P values as exact values whenever suitable.*
☒	☐	For Bayesian analysis, information on the choice of priors and Markov chain Monte Carlo settings
☒	☐	For hierarchical and complex designs, identification of the appropriate level for tests and full reporting of outcomes
☒	☐	Estimates of effect sizes (e.g. Cohen's *d*, Pearson's *r*), indicating how they were calculated

Our web collection on statistics for biologists contains articles on many of the points above.

Software and code

Policy information about availability of computer code

Data collection	RNA-seq, CUT&Tag-seq and ATAC-seq data are collected at home by using the specific commercial kit (Vazyme, NR606, TD903 and TD501).
Data analysis	RNA-seq data were mapped to the mm10 reference genome with HISAT2 (v2.2.1) and Trim Galore (v0.6.4). StringTie (v2.2.1) was used to do quantification. GFOLD (v1.1.4) was used to perform differential expression analysis between conditions. The ATAC-seq and CUT&Tag seq were mapped to the mm10 reference genome using bowtie2 (v2.4.5) and Trim Galore (v0.6.4). SAMtools (v1.16.1) and MACS (v1.4.2) for quantification and peak calling. clusterProfiler (v4.6.2) for Gene Ontology analysis. HOMER (v4.11.1) for motif analysis. Oct4-GFP positive colonies were counted using Image-J software (v1.54f, NIH). MaxQuant version 1.6.0.1 for mass spectrometry data. FlowJo v.7.6.1. for flow cytometry. The code generated in this study is available on GitHub [https://github.com/LonlyWinter/eCR_Code].

For manuscripts utilizing custom algorithms or software that are central to the research but not yet described in published literature, software must be made available to editors and reviewers. We strongly encourage code deposition in a community repository (e.g. GitHub). See the Nature Portfolio guidelines for submitting code & software for further information.

Data

Policy information about availability of data

All manuscripts must include a data availability statement. This statement should provide the following information, where applicable:

- Accession codes, unique identifiers, or web links for publicly available datasets
- A description of any restrictions on data availability
- For clinical datasets or third party data, please ensure that the statement adheres to our policy

The RNA-Seq, CUT&Tag, ATAC-seq data generated in this study have been deposited in the Gene Expression Omnibus database under the accession number GSE243517 [https://www.ncbi.nlm.nih.gov/geo/query/acc.cgi?acc=GSE243517]. The code generated in this study is available on GitHub [https://github.com/LonlyWinter/eCR_Code]. The mass spectrometry proteomics data have been deposited to the ProteomeXchange Consortium via the iProX partner repository with the dataset identifier PXD046008 [https://proteomecentral.proteomexchange.org/cgi/GetDataset?ID=PXD046008]. All other data generated in this study are available in the Supplementary Information or Source data file.

Research involving human participants, their data, or biological material

Policy information about studies with human participants or human data. See also policy information about sex, gender (identity/presentation), and sexual orientation and race, ethnicity and racism.

Reporting on sex and gender	*Use the terms sex (biological attribute) and gender (shaped by social and cultural circumstances) carefully in order to avoid confusing both terms. Indicate if findings apply to only one sex or gender; describe whether sex and gender were considered in study design; whether sex and/or gender was determined based on self-reporting or assigned and methods used. Provide in the source data disaggregated sex and gender data, where this information has been collected, and if consent has been obtained for sharing of individual-level data; provide overall numbers in this Reporting Summary. Please state if this information has not been collected. Report sex- and gender-based analyses where performed, justify reasons for lack of sex- and gender-based analysis.*
Reporting on race, ethnicity, or other socially relevant groupings	*Please specify the socially constructed or socially relevant categorization variable(s) used in your manuscript and explain why they were used. Please note that such variables should not be used as proxies for other socially constructed/relevant variables (for example, race or ethnicity should not be used as a proxy for socioeconomic status). Provide clear definitions of the relevant terms used, how they were provided (by the participants/respondents, the researchers, or third parties), and the method(s) used to classify people into the different categories (e.g. self-report, census or administrative data, social media data, etc.) Please provide details about how you controlled for confounding variables in your analyses.*
Population characteristics	*Describe the covariate-relevant population characteristics of the human research participants (e.g. age, genotypic information, past and current diagnosis and treatment categories). If you filled out the behavioural & social sciences study design questions and have nothing to add here, write "See above."*
Recruitment	*Describe how participants were recruited. Outline any potential self-selection bias or other biases that may be present and how these are likely to impact results.*
Ethics oversight	*Identify the organization(s) that approved the study protocol.*

Note that full information on the approval of the study protocol must also be provided in the manuscript.

Field-specific reporting

Please select the one below that is the best fit for your research. If you are not sure, read the appropriate sections before making your selection.

☒ Life sciences ☐ Behavioural & social sciences ☐ Ecological, evolutionary & environmental sciences

For a reference copy of the document with all sections, see nature.com/documents/nr-reporting-summary-flat.pdf

Life sciences study design

All studies must disclose on these points even when the disclosure is negative.

Sample size	No sample size calculation was performed. Sample size was chosen based on our experience and experiments. No statistical method was used to predetermine sample size.
Data exclusions	No data were excluded from analysis
Replication	All experiments were replicated at least three times, data are shown as means with SD.
Randomization	The authors thought there were no relevant for randomization to our study. No statistical method was used to predetermine sample size. No specific randomization or blinding protocols were used. Each experiment had positive and negative controls, and the sample size was carefully designed to support the conclusions.

Blinding	None

Reporting for specific materials, systems and methods

We require information from authors about some types of materials, experimental systems and methods used in many studies. Here, indicate whether each material, system or method listed is relevant to your study. If you are not sure if a list item applies to your research, read the appropriate section before selecting a response.

Materials & experimental systems

n/a	Involved in the study
☐	☒ Antibodies
☐	☒ Eukaryotic cell lines
☒	☐ Palaeontology and archaeology
☐	☒ Animals and other organisms
☒	☐ Clinical data
☒	☐ Dual use research of concern
☒	☐ Plants

Methods

n/a	Involved in the study
☐	☒ ChIP-seq
☐	☒ Flow cytometry
☒	☐ MRI-based neuroimaging

Antibodies

Antibodies used	anti-NANOG(Abcam, ab214549, 1:1000), anti-FLAG (Sigma, F1804, 1:1000), anti-SMARCA4 (Abcam, ab110641, 1:5000), anti-SMARCC1 (Abcam, ab305037, 1:1000) for western blot. Alexa fluor 647 anti-SSEA-1 (Biolegend, 125607, 1:100), HRP-labeled Goat Anti-Rabbit IgG(H+L) (Beyotime, A0208, 1:5000) , HRP-labeled Goat Anti-Mouse IgG(H+L) (Beyotime, A0216, 1:5000) for flow cytometry. Antibodies against NANOG (Abcam, ab214549, 1:50), H3K4me1 (Abcam, ab8895, 1:50), H3K27ac (Abcam, ab4729, 1:50), flag (Sigma, F1804, 1:50), and SMARCA4 (Cell Signaling Technology, 52251, 1:50), Goat anti-Rabbit IgG(H+L) (Abcam, ab6702, 1:200), Goat anti-Mouse IgG(H+L) (Abcam, ab6708, 1:200) for CUT&Tag seq.
Validation	anti-NANOG: https://www.abcam.cn/products/primary-antibodies/nanog-antibody epr20694-chip-grade-ab214549.html<br>anti-FLAG: https://www.sigmaaldrich.cn/CN/zh/product/sigma/f1804<br>anti-SMARCA4: https://www.abcam.cn/products/primary-antibodies/brg1-antibody-epncir111a-ab110641.html<br>anti-SMARCC1: https://www.abcam.cn/products/primary-antibodies/smarcc1baf155-antibody-epr25109-77-ab305037.html<br>Alexa fluor 647 anti-SSEA-1: https://www.biolegend.com/en-us/products/alexa-fluor-647-anti-mouse-human-cd15-ssea-1-antibody-4819<br>anti-H3K4me1: https://www.abcam.cn/products/primary-antibodies/histone-h3-mono-methyl-k4-antibody-chip-grade-ab8895.html<br>anti-H3K27ac: https://www.abcam.cn/products/primary-antibodies/histone-h3-acetyl-k27-antibody-chip-grade-ab4729.html<br>anti-SMARCA4: https://www.cellsignal.com/products/primary-antibodies/brg1-e9o6e-mouse-mab/52251<br>Goat anti-Rabbit IgG(H+L): https://www.abcam.cn/products/secondary-antibodies/goat-rabbit-igg-hl-ab6702.html<br>Goat anti-Mouse IgG(H+L): https://www.abcam.cn/products/secondary-antibodies/goat-mouse-igg-hl-ab6708.html<br>HRP-labeled Goat Anti-Rabbit IgG(H+L) https://www.beyotime.com/product/A0208.html<br>HRP-labeled Goat Anti-Mouse IgG(H+L) https://www.beyotime.com/product/A0216.html

Eukaryotic cell lines

Policy information about cell lines and Sex and Gender in Research

Cell line source(s)	OG2 MEFs were isolated from E13.5 embryos (female or male) by crossing male OG2 mice to 129 female mice.<br>Mouse neonatal fibroblasts (MNFs) were isolated from dorsal skin dermis of day2-3 neonatal pups (female or male). Mouse tail tip fibroblasts (TTFs) were isolated from 8- week old mice (female or male). Plat-E cells were obtained from Guangzhou institutes of biomedicine and healthy, Chinese academy of sciences. Mouse ESCs were derived from embryos (female or male) of OG2 mice and iPSCs were derived from this study.
Authentication	All the cell line used were authenticated
Mycoplasma contamination	All cell lines were tested negative for mycoplasma with the Kit from Lonza (LT07-318).
Commonly misidentified lines (See ICLAC register)	None

Animals and other research organisms

Policy information about studies involving animals; ARRIVE guidelines recommended for reporting animal research, and Sex and Gender in Research

Laboratory animals	Oct4-GFP (OG2) reporter-allele-carrying mice (CBA/CaJ x C57BL/6J, 7-8 weeks, male and female) were obtained from The Jackson Laboratory. The wild-type female 129 mice (129S2/SvPasCrl, 7-8 weeks) and wild-type ICR mice (ICRCF1/Crl, 7-8 weeks, male and female) were purchased from Vital River Laboratory Animal Technology Co., Ltd (Beijing). All animal were housed in a temperature-controlled room with a 12-hour light/dark cycle, 20?-26? ambient temperature and 40%-70% humidity. Diet and water were available ad libitum.
Wild animals	None
Reporting on sex	None
Field-collected samples	This study did not involve samples collected from the field.
Ethics oversight	The animal studies were performed according to the applicable guidelines and regulations of Institutional Animal Care and Use Committee (IACUC) of Westlake University (Animal Protocol No. 23-107-PDQ), Hangzhou, China.

Note that full information on the approval of the study protocol must also be provided in the manuscript.

Plants

Seed stocks	*Report on the source of all seed stocks or other plant material used. If applicable, state the seed stock centre and catalogue number. If plant specimens were collected from the field, describe the collection location, date and sampling procedures.*
Novel plant genotypes	*Describe the methods by which all novel plant genotypes were produced. This includes those generated by transgenic approaches, gene editing, chemical/radiation-based mutagenesis and hybridization. For transgenic lines, describe the transformation method, the number of independent lines analyzed and the generation upon which experiments were performed. For gene-edited lines, describe the editor used, the endogenous sequence targeted for editing, the targeting guide RNA sequence (if applicable) and how the editor was applied.*
Authentication	*Describe any authentication procedures for each seed stock used or novel genotype generated. Describe any experiments used to assess the effect of a mutation and, where applicable, how potential secondary effects (e.g. second site T-DNA insertions, mosiacism, off-target gene editing) were examined.*

ChIP-seq

Data deposition

☒ Confirm that both raw and final processed data have been deposited in a public database such as GEO.

☒ Confirm that you have deposited or provided access to graph files (e.g. BED files) for the called peaks.

Data access links<br>*May remain private before publication.*	All sequencing data that support the findings of this study have been deposited in the Gene Expression Omnibus database under the accession number GSE243517[https://www.ncbi.nlm.nih.gov/geo/query/acc.cgi?acc=GSE243517]
Files in database submission	HT-T-26: NanogBiD+Oct4, H3K4me, Day5<br>HT-T-27: NanogBiD+Oct4, H3K27ac, Day5<br>HT-T-28: NanogBiD+Oct4, Smarca4 (Brg1), Day5<br>HT-T-30: NanogBiD+Oct4, Flag, Day5<br>HT-T-32: Nanog+Oct4, Flag, Day5<br>HT-T-33: Nanog+Oct4, H3K4me, Day5<br>HT-T-34: Nanog+Oct4, Smarca4 (Brg1), Day5<br>HT-T-35: Nanog+Oct4, H3K27ac, Day5<br>HT-C-52: Nr5a2BiD<br>HT-C-53: Nr5a2<br>HT-C-54: Oct4BiD<br>HT-C-55: Oct4<br>HT-C-56: Sox2BiD<br>HT-C-57: Sox2<br>HT-C-60: NanogBiD<br>HT-C-61: Nanog
Genome browser session<br>(e.g. UCSC)	no longer applicable

Methodology

Replicates	There are no biological replications. The CUT&Tag experiment was performed once.

Sequencing depth

SampleName Layout TotalPairs UnmappedPairs UniqMappedPairs MultiMappedPairs DiscordantlyAlignedPairs UnpairedReads UnmappedReads UniqMappedReads MultiMappedReads OverallAlignmentRate UniqAlignmentRate
Nr5a2BiD pair 42070872 4854311 33228085 3988476 2939104 3830414 2177672 960219 692523 97.41% 78.98%
Nr5a2 pair 34862315 3799423 28827777 2235115 1994142 3610562 2212050 1020514 377998 96.83% 82.69%
Oct4BiD pair 29842264 2667244 25370769 1804251 1224643 2885202 1762692 837591 284919 97.05% 85.02%
Oct4 pair 37066795 4151415 28995123 3920257 2218649 3865532 2338199 879790 647543 96.85% 78.22%
Sox2BiD pair 34445307 3128553 29520750 1796004 1803467 2650172 1531090 798750 320332 97.78% 85.70%
Sox2 pair 50681877 4542665 38864811 7274401 2209664 4666002 2855877 899311 910814 97.18% 76.68%
NanogBiD pair 36150114 4438809 29812374 1898931 2753007 3371604 1778485 1127058 466061 97.54% 82.47%
Nanog pair 30181354 3865526 23926541 2389287 2256140 3218772 1814859 879248 524665 96.99% 79.28%
NanogBiD.Oct4.Flag pair 11068132 2154754 8115620 797758 1505780 1297948 625587 382463 289898 97.17% 73.32%
NanogBiD.Oct4.Brg1 pair 35854678 6700214 26500945 2653519 4635491 4129446 1817006 1294788 1017652 97.47% 73.91%
Nanog.Oct4.Brg1 pair 15330006 2244810 11818074 1267122 1439439 1610742 820284 444626 345832 97.32% 77.09%
Nanog.Oct4.H3K4me pair 45853878 5312050 38293078 2248750 3753055 3117990 1476899 1096945 544146 98.39% 83.51%
NanogBiD.Oct4.H3K4me pair 32630211 5485391 25667352 1477468 4170313 2630156 1177966 890707 561483 98.19% 78.66%
Nanog.Oct4.H3K27ac pair 43900956 7229926 33820120 2850910 5155864 4148124 1758435 1391028 998661 98.00% 77.04%
NanogBiD.Oct4.H3K27ac pair 40924420 9131211 29496084 2297125 6871876 4518670 1927620 1395798 1195252 97.64% 72.07%
Nanog.Oct4.Flag pair 1835854 283723 1425517 126614 202817 161812 72823 49471 39518 98.02% 77.65%

Antibodies

Antibodies against NANOG (Abcam, ab214549, 1:50), H3K4me1 (Abcam, ab8895, 1:50), H3K27ac (Abcam, ab4729, 1:50), FLAG (Sigma, F1804, 1:50), and SMARCA4 (Cell Signaling Technology, 52251, 1:50), Goat anti-Rabbit IgG(H+L) (Abcam, ab6702, 1:200), Goat anti-Mouse IgG(H+L) (Abcam, ab6708, 1:200)

Peak calling parameters

The default cutoff of MACS were used for the significantly enriched peaks.

Data quality

SAMtools (v1.16.1) was used to remove the repetitive, low sequencing quality (mapq<30) and the mitochondrial DNA mapped reads in the total mapped reads.

Software

Trim Galore (v0.6.4) , bowtie2 (v2.4.5), SAMtools (v1.16.1), MACS (v1.4.2), HOMER (v4.11.1)

Flow Cytometry

Plots

Confirm that:

☒ The axis labels state the marker and fluorochrome used (e.g. CD4-FITC).

☒ The axis scales are clearly visible. Include numbers along axes only for bottom left plot of group (a 'group' is an analysis of identical markers).

☒ All plots are contour plots with outliers or pseudocolor plots.

☒ A numerical value for number of cells or percentage (with statistics) is provided.

Methodology

Sample preparation

Cells were dissociated with 0.05% trypsin-EDTA. After washed by PBS, cells were blocked by PBS supplemented with 1% FBS, followed by incubation with antibody against SSEA-1 (Biolegend, 125607, 1:100) for 1 h at 4°C. After centrifugation, cells were washed twice with PBS, resuspended with PBS containing 0.1% BSA, filtered using a cell strainer (BD Biosciences).

Instrument

CytoFLEX 6L flow cytometer (BD Biosciences)

Software

Data analysis was performed using FlowJo v.7.6.1.

Cell population abundance

A minimum of 10000 cells were counted per sample analyzed.

Gating strategy

we set the preliminary FSC/SSC gate to remove debris, and single cell gate to select for single cells, then we set the gate based on the fluorescence negative control. Please see an example in supplementary Figure 2.

☒ Tick this box to confirm that a figure exemplifying the gating strategy is provided in the Supplementary Information.
